# Assessment of Brazilian children’s anxiety during the second year of the COVID-19 pandemic through cross-sectional interviews at schools

**DOI:** 10.3389/fpubh.2025.1522105

**Published:** 2025-05-12

**Authors:** Patrícia Aparecida Francelino Crepalde, Marla Andréia Garcia de Avila, Michelle Cristine de Oliveira Minharro, Meire Cristina Novelli Castro, Tatiane Roberta Fernandes Teixeira, Pedro Tadao Hamamoto Filho, Stefan Nilsson

**Affiliations:** ^1^Department of Nursing, Botucatu Medical School– São Paulo State University - UNESP-, Botucatu, Brazil; ^2^Department of Neurosciences and Mental Health, Botucatu Medical School– São Paulo State University - UNESP, Botucatu, Brazil; ^3^Institute of Health and Care Sciences, Sahlgrenska Academy, University of Gothenburg, Gothenburg, Sweden; ^4^Centre for Person-Centred Care, Sahlgrenska Academy, University of Gothenburg, Gothenburg, Sweden; ^5^The Queen Silvia Children’s Hospital, Gothenburg, Sweden

**Keywords:** children, anxiety, pandemic, COVID-19, nursing

## Abstract

**Introduction:**

This cross-sectional study aimed to assess anxiety prevalence among schoolchildren and associated factors following their return to in-person classes during the COVID-19 pandemic.

**Methods:**

This study was conducted in June 2022 and involved children aged 6–12 years and their parents at four public schools in Brazil. Anxiety levels were assessed using the Children’s Anxiety Questionnaire (CAQ; scores of 4–12) and visual analog scale (VAS; scores of 0–10). Of 733 children, 54% were girls (average age, 8.7 ± 1.87 years), and most of the included parents were mothers (84%).

**Results:**

Based on Brazilian CAQ scores of ≥9 and VAS scores of >7, anxiety prevalence was 13.5 and 6.7%, respectively. Using logistic regression, CAQ scores of ≥9 and VAS scores of >7 were associated with the children’s ages. Each additional year of children’s age raised the odds of anxiety (CAQ ≥ 9) by 10%. For the VAS scores, each additional year of children’s age increased the odds of anxiety (VAS > 7) by 30%.

**Discussion:**

These findings showed a lower prevalence than those reported at the beginning and one year after the start of the COVID-19 pandemic, further emphasizing the need for public health interventions targeting different age groups.

## 1 Introduction

The coronavirus disease 2019 (COVID-19) pandemic lasted longer than expected and has resulted in numerous studies investigating its impact on the mental health of children and adolescents (1–5). Furthermore, the prevalence of mental health symptoms increased as the pandemic progressed, and studies showed different associated factors (1–5). Racine et al.’s 2021 global meta-analysis, which included 29 studies conducted between January 2020 and March 2021 and involved 80,879 participants, revealed that the prevalence of anxiety and depression among children and adolescents during the first year of the pandemic was 20.5 and 25.2%, respectively. The researchers noted that the pooled estimates were twice that of pre-pandemic estimates. Panchal et al. conducted a global meta-analysis of 74 studies that included 478,882 participants (mean age, 13.4 years; 52.3% female) (3, 6). In this study, the pooled rate of children and adolescents fulfilling the diagnostic criteria for anxiety disorders was 13.0%, and the pooled prevalence of anxiety symptoms was 26.5%. Anxiety symptoms were significantly more prevalent in boys than in girls, significantly higher during the second wave of the pandemic (after July 2020) than the first wave (before June 2020), and more prevalent during school closure.

Deng et al.’s meta-analysis of 191 studies, which included 1,389,447 children and adolescents, reported that, during the COVID-19 pandemic, the pooled prevalence of depressive symptoms was 31% based on 129 studies (*n* = 524,417), and the prevalence of mild, moderate, and severe depressive symptoms was 19, 13, and 6%, respectively (1). The pooled prevalence of sleep disturbances from 50 studies (*n* = 104,219) was 42%. Age, grade level, education level, sex, geographical region, and electronic device use were associated with an increased prevalence of mental health symptoms. The prevalence of mental health symptoms also increased as the pandemic progressed, although signs of recovery and stabilization were observed (1).

A systematic review and meta-analysis that included 868,634 children and adolescents (≤19 years) pre-pandemic and 807,480 during the COVID-19 pandemic in Europe compared depression symptoms during the pre-pandemic vs. pandemic periods. The increase in general depression symptoms was higher for male adolescents, whereas the increase in clinically relevant depression rates was higher for females. Effect estimates were significantly higher when pandemic-related restrictions were more stringent or when school closures occurred (7).

Children are more vulnerable during crises, such as a pandemic, due to their lack of autonomy, poor maturity, and low influence in society. During the pandemic, anxiety and depressive symptoms increased considerably among individuals, suggesting the need for continuous mental health monitoring. However, limited research has examined school return after an absence due to school closures, especially in middle-and low-income countries (1–5). Therefore, this study aimed to assess the prevalence of anxiety among school children aged 6–12 years following their return to in-person classes during the COVID-19 pandemic. Furthermore, we aimed to examine factors associated with children’s anxiety following school return.

## 2 Methods

### 2.1 Study design

A cross-sectional study using nonprobability and convenience sampling methods was conducted in June 2022 across four public schools. We followed the design utilized in a similar study that assessed anxiety levels at the onset of the COVID-19 pandemic (2, 8) and adapted this design to collect data in a face-to-face manner.

Data reporting followed the recommendations of the Strengthening the Reporting of Observational Studies in Epidemiology (STROBE) statement to ensure the quality of the study methods (9).

During the data collection period, school disclosure was categorized as level 4, indicating that it was required at all levels (10). Since August 2021, schools have returned to in-person classes, using preventive measures such as wearing masks and hand hygiene.

### 2.2 Participants

The study was conducted in Querência, a city in the northeast of the Mato Grosso state. In total, 2,574 school students aged 5–15 years were enrolled in the 4 schools as follows: Schools A (*n* = 424), B (*n* = 458), C (*n* = 813) and D (*n* = 879). Of those students, 825 children aged between 6 and 12 years were eligible for the study.

The participants included schoolchildren aged 6–12 years from four public schools in Querência, Cuiabá Capital, and their parents or guardians. We excluded parents under the age of 18 years, those who were illiterate, and those who did not respond to the majority of the parental questionnaire. We also excluded children who were absent on the day of data collection.

Two researchers collected data. The parents’ and children’s questionnaires were self-administered in the presence of the researchers.

### 2.3 Assessments of anxiety

The Children’s Anxiety Questionnaire (CAQ), with scores ranging from 4 to 12, and the visual analog scale (VAS), with scores ranging from 0 to 10, were used to measure anxiety levels among the participating children. The CAQ, developed in Sweden and available in English, Swedish (11, 12), and Portuguese (13), was based on the State–Trait Anxiety Inventory (14). The CAQ contained four items with four facial expression images and three response options, each representing a different level of emotional intensity (11, 13). Children responded based on the four facial expressions, one at a time, and picked one of three responses (i.e., a little, 1; some, 2; a lot, 3). The faces of happy/content and calm/relaxed were measured as 3–2–1, whereas those of tense/nervous and worried/afraid were measured as 1–2–3. The minimum aggregate score was 4, representing the lowest anxiety level for all four items. The CAQ in Brazilian Portuguese was recently validated and demonstrated satisfactory results among professionals and children (13), with adequate psychometric properties (15).

The self-reported VAS was used; in this scale, 0 (zero) equaled “calm,” and 10 equaled “very anxious.” Despite the VAS showing a high correlation with the State–Trait Anxiety Inventory (r = 0.55; *p* < 0.01) and its usage in various studies, its psychometric properties require further investigation (16, 17).

Following previous research, the cut-off value for the CAQ was set at the levels of <9 or ≥9 for low and high, respectively. For the VAS, the cut-off value was set at the levels of ≤7 or >7 for low and high, respectively (8).

### 2.4 Data collection

A brief description of the study and its objectives were provided to the children’s parents and guardians. They completed the parental form at home and provided authorization for researchers to conduct face-to-face interviews with their children at school. With the children, the researcher collected data in each class, assisting them in completing the CAQ, VAS, and open questions following the survey instructions.

The survey examined the sociodemographic profiles and current conditions of the children and their parents. The survey comprised 16 closed items. The quantitative variables for the parents included sex (female or male), age, education level (elementary school, high school, college, or postgraduate degree), relationship with the child (mother, father, and others), whether parents were healthcare professionals (yes or no), confirmation of COVID-19 diagnoses among immediate family members (yes or no), and income reductions during the pandemic (yes or no). The variables measured for the children included whether the child had received the COVID-19 vaccine (first or second dose, yes or no), whether the child had a chronic disease or disability (yes or no), details of any chronic disease or disability (open question), whether the child was identified as Indigenous (yes or no), the child’s sex (female or male), age, and whether the child participated in leisure activities during the pandemic (yes or no). At school, children completed information on which school they attended (A, B, C, or D) and provided individual responses to the CAQ and VAS, as well as answers to four open questions: “Tell me what makes you happy/content, calm/relaxed, tense/nervous, or worried/afraid.”

### 2.5 Sample and statistical analysis

Brazil has approximately 27,424,401 children aged 5–14 years (18). Consequently, we employed a non-probability sampling method, specifically convenience sampling, to gather the necessary information.

The Shapiro–Wilk test was used to evaluate the distribution of continuous data. Comparisons of continuous data between groups were performed using the Mann–Whitney U test or Student’s t-test for non-parametric and parametric data. For comparisons involving multiple groups, the Kruskal–Wallis test followed by Dunn’s tests or one-way analysis of variance (ANOVA) followed by Tukey’s test were performed. Spearman’s rank (rho) correlation coefficients were calculated between the CAQ and VAS scores. Proportions in different groups were compared using the chi-squared (for *n* > 30) or Fisher’s exact tests. Odds ratios (ORs) were calculated to evaluate the association between outcome variables, such as the dependent variable (VAS or CAQ scores; as binary categories defined as <9 or ≥9 and ≤7 or >7, respectively).

A logistic regression analysis was performed to evaluate the associations between high or low anxiety scores (≥9 for CAQ and >7 for VAS scores) and various independent variables. Independent variables with a *p*-value < 0.10 in the univariate analysis for either CAQ or VAS scores were included in the logistic models. Statistical significance was set at *p* < 0.05. All statistical analyses were performed using IBM SPSS Statistics for MacBook version 24 (IBM Corp., Armonk, NY, USA). Finally, the point prevalence of anxiety was compared with a previous survey, using confidence intervals (CIs) set at 95% and the corresponding ORs.

### 2.6 Ethical information

This study was approved in June 2022 by the appropriate ethics review board (CAAE: 58609022.0.0000.5411; opinion number: 5.444.291).

## 3 Results

In total, 825 children were eligible, and their parents were invited to a meeting with the researchers. Parents who attended the meeting (*n* = 792) with the researchers were invited to access the data collection instrument at home. Of these, 59 were excluded for not meeting the inclusion criteria, resulting in a final sample of 733 parents and their children.

Among the children, the mean age was 8.7 ± 1.87 years, with the majority being girls 54% (*n* = 396). Of the children, 7.4% (*n* = 54) had chronic diseases or disabilities, with asthma/bronchitis being the most common (3%, *n* = 22; Table 1). Based on a CAQ score of ≥9 and a VAS score of >7, the anxiety prevalence was 13.5 and 6.7%, respectively.

**Table 1:** Sociodemographic and COVID-19-related characteristics of the children and their parents.

Variable	N	%
Child’s sex		
Female	396	54.0
Male	337	46.0
Age, mean ± SD (years)	8.7 ± 1.87	
School		
A	193	26.3
B	317	43.2
C	180	24.6
D	43	5.9
Has a chronic disease or disability (yes)	54	7.4
COVID-19 vaccination (yes)	278	37.9
CAQ score, median (range)	7.15 (4–12)	
VAS score, median (range)	5.98 (0–10)	
Parents’ age, mean ± SD (years)	34.2 ± 6.8	
Income decreased during pandemic (yes)	250	34.1
Health professionals COVID-19 (yes)	33	4.5
Parental relationship*		
Mother	616	84.0
Father	86	11.7
Other family member	5	0.7
Missing	26	
Parents’ schooling level		
Post-graduate	60	8.2
Graduate	135	18.4
High school	357	48.7
Elementary school	181	24.7
COVID-19 death in a family member (yes)	44	6.0
Confirmed COVID-19 in family member (yes)	312	42.6

Querência, MT, Brazil, 2022.

COVID-19, coronavirus disease 2019; CAQ, children’s anxiety questionnaire; VAS, visual analog scale; SD, standard deviation.

The mean age of the included parents was 34.2 ± 6.8 years, with the majority being mothers (84%; *n* = 616). Among the parents, 357 (48.7%) had completed at least high school, and 4.5% (*n* = 33) were healthcare professionals (Table 1).

Table 2 shows the breakdown of children’s open responses following the CAQ items.

**Table 2:** Breakdown of children’s open responses.

Open question	*N*	%
Tell me what makes you happy and content (*n* = 687)		
Family/mother/father	223	32.5
To play	166	24.2
Leisure activities (reading, listening to music, and drawing)	105	15.3
Being at school	48	7.0
Food	47	6.8
Being with friends	22	3.2
Electronics	15	2.2
Others	61	8.9
Tell me what makes you calm and relaxed (*n* = 765)		
To sleep/relax	148	19.3
Family/mother/father	129	16.9
Electronics	117	15.3
Leisure activities (reading, listening to music, and drawing)	104	13.6
To play	51	6.7
Food	34	4.4
Affection, love	30	3.9
School	19	2.5
Being with friends	18	2.4
Pets/animals	17	2.2
Others	98	12.8
Tell me what makes you tense and nervous (*n* = 641)		
Frustrations	186	29.0
A person arguing with me	81	12.6
My brother or my sister	79	12.3
Homework	54	8.4
Closed place	46	7.2
Illness/medication	38	5.9
Schoolwork	17	2.7
Friends	14	2.2
To see dangerous animals	11	1.7
Others	115	17.9
Tell me what makes you worried and afraid (*n* = 703)		
My family, parents, or pets dying	161	22.9
Dark places	75	10.7
Getting sick/getting hurt	67	9.5
Being alone	66	9.4
Animals	48	6.8
Doing homework	38	5.4
My parent’s recommendations	33	4.7
Frustrations	28	4.0
Fights	18	2.6
Others	169	24.0

Querência, MT, Brazil, 2022.

**Figure 1:**
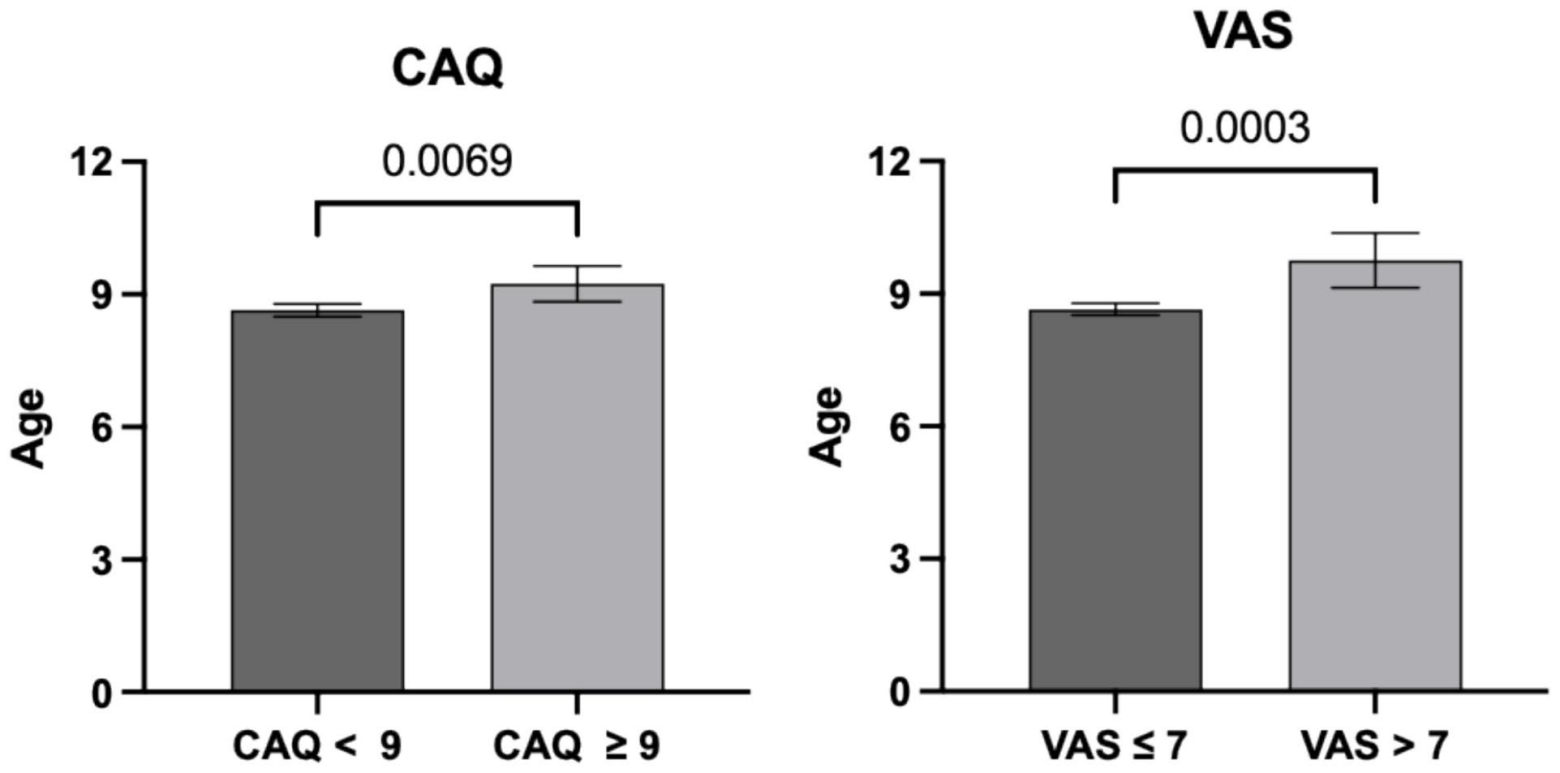
Children ages according to the presence of anxiety (CAQ ≥ 9 or VAS > 7). Children with anxiety were older than those without anxiety. *p*-values are presented above the brackets.

Children’s age was different according to the presence of anxiety: for CAQ, 8.6 ± 1.8 vs. 9.2 ± 2.0 years for children without and with anxiety (*p* = 0.007), respectively. For VAS, 8.7 ± 1.8 vs. 9.8 ± 2.2 years for children without and with anxiety (*p* < 0.001), respectively (Figure 1).

Table 3 shows the prevalence of anxiety according to instruments and their association with each variable; variables associated with CAQ or VAS scores are shown in bold. The prevalence of anxiety was higher for children of senior levels in both scales (for CAQ, *p* = 0.01; for VAS, *p* = 0.001).

**Table 3:** Bivariate analyses between anxiety (CAQ score ≥9; VAS score >7) and the main characteristics of the population.

	CAQ (*n* = 99/733)	%	*p*-value	VAS (*n* = 49/733)	%	*p*-value
Schools						
A (*n* = 193)	36	18.7		24	12.4	
B (*n* = 317)	36	11.4		15	4.7	
C (*n* = 180)	21	11.7	0.104	9	5.0	**0.003**
D (*n* = 43)	6	14.0		1	2.3	
Class school						
First year (*n* = 119)	12	10.1		3	2.5	
Second year (*n* = 142)	17	12.0		8	5.6	
Third year (*n* = 99)	13	13.1		5	5.1	
Fourth year (*n* = 90)	7	7.8	**0.01**	2	2.2	**<0.001**
Fifth year (*n* = 164)	23	14.0		12	7.3	
Sixth year (*n* = 65)	11	16.9		7	10.8	
Seventh year (*n* = 54)	16	29.6		12	22.2	
Parental relationship						
Mother (*n* = 616)	83	13.5		39	6.3	
Father (*n* = 86)	11	12.8	0.896	7	8.1	0.653
Others (*n* = 31)	5	16.1		3	9.7	
Parents schooling levels						
Elementary school (*n* = 181)	33	18.2		17	9.4	
High school (*n* = 357)	39	10.9	0.085	21	5.9	0.311
Graduate (*n* = 135)	21	15.6		9	6.7	
Post-graduate (*n* = 60)	6	10.0		2	3.3	
Parents jobs						
Healthcare professionals (*n* = 33)	4	12.1		2	6.1	
Others (*n* = 617)	75	12.2		36	5.8	
Missing (*n* = 79)	18	22.8	**0.016**	10	12.7	0.065
Unemployed (*n* = 4)	2	50.0		1	25.0	
Parent’s income reduced during pandemic						
Yes (*n* = 250)	42	16.8		17	6.8	
No (*n* = 436)	48	11.0	0.052	29	6.7	0.394
Missing (*n* = 47)	9	19.1		3	6.4	
Indigenous children						
Yes (*n* = 12)	1	8.3	0.84	1	8.3	1
Children received COVID-19 vaccinations						
One dose (*n* = 114)	16	14.0		7	6.1	
Two doses (*n* = 164)	30	18.3	0.101	14	8.5	0.587
None (*n* = 455)	53	11.6		28	6.2	
Had a chronic disease or disability						
Yes (*n* = 54)	6	11.1	0.587	1	1.9	0.166
Diagnoses of COVID-19 among immediate family members						
Yes (*n* = 312)	46	14.7	0.399	18	5.8	0.393
Death from COVID-19 among immediate family members						
Yes (*n* = 43)	5	11.6	0.710	1	2.3	0.351
Leisure activities during the pandemic						
Yes (*n* = 62)	7	11.3	0.594	5	8.1	0.790

Querência, MT, Brazil, 2022.

COVID-19, coronavirus disease 2019; CAQ, Children’s Anxiety Questionnaire; VAS, visual analog scale. ^1^*p*-value for comparisons with CAQ; ^2^*p*-value for comparisons with VAS. Significance value at *p* < 0.05 in bold.

The binary logistic regression for the CAQ scores showed that with each additional year of children’s age, the risk of anxiety (CAQ ≥ 9) increased by 10% (Table 4). Also, for CAQ, reduced income was associated with higher anxiety scores (*p* = 0.022). Moreover, the logistic regression for the VAS scores showed that with each additional year of children’s age, the risk of anxiety (VAS > 7) increased by 30% (Table 5). In the multivariate analysis, only the child’s age remained associated with anxiety on the CAQ and VAS scores (*p* = 0.005 and *p* < 0.001, respectively).

**Table 4:** Logistic regression analysis for anxiety based on CAQ scores (≥9 vs. <9).

Independent variable	OR	95% CI	*p*-value
Child’s age	1.1	1.052–1.327	**0.005**
Elementary school level	Ref	Ref	
High school level	0.6	0.372–1.057	0.080
Graduate level	1.0	0.537–1.860	0.999
Post-graduate level	0.6	0.239–1.583	0.313
Unemployed	Ref	Ref	
Healthcare professional	0.1	0.019–1.741	0.139
Non-healthcare professional	0.1	0.021–1.157	0.069
No reduced income	Ref	Ref	
Reduced income	1.7	1.082–2.718	**0.022**

Querência, MT, Brazil, 2022.

OR, odds ratio; CI, confidence interval; CAQ, children’s anxiety questionnaire. Significance value at *p* < 0.05 in bold.

**Table 5:** Logistic regression analysis for anxiety based on VAS scores (>7 vs. ≤7).

Independent variable	OR	95% CI	*p*-value
Child’s age	1.3	1.158–1.595	**<0.001**
Elementary school level	Ref	Ref	
High school level	0.7	0.362–1.450	0.080
Graduate level	0.8	0.336–1.915	0.619
Post-graduate level	0.3	0.080–1.667	0.193
Unemployed	Ref	Ref	
Healthcare professional	0.3	0.019–4.776	0.397
Non-healthcare professional	0.2	0.025–2.797	0.269
No reduced income	Ref	Ref	
Reduced income	1.0	0.570–2.047	0.812

Botucatu, SP, Brazil, 2022.

OR, odds ratio; CI, confidence interval; VAS, visual analog scale.

Significance value at *p* < 0.05 in bold.

## 4 Discussion

This study provides critical insights into mental health symptoms among students during the return to in-person schooling in Brazil after lockdown during the COVID-19 pandemic. Additionally, using diverse data collection methods, including surveys and interviews, adds depth to the understanding of children’s experiences and feelings, offering a nuanced perspective on the issue.

Our findings revealed decreased anxiety rates when compared to similar previous research conduct in Brazil (2, 8, 19). In particular, the anxiety rates after the beginning of the COVID-19 pandemic and with the return to in-person classes among children aged two years were 13.5 and 6.7%, according to the CAQ and VAS scores, respectively. These results are similar to the prevalence of anxiety reported for Brazilian children under normal conditions (12.7%) (20). Conversely, Garcia de Avila et al. investigated the prevalence of anxiety among 289 children in Brazil aged 6–12 years at the onset of the pandemic and reported anxiety rates of 19.4% (*n* = 56) using CAQ scores and 21.8% (*n* = 63) using the numerical rating scale (NRS). In a follow-up study 1 year into the pandemic including 906 children aged 6–12 years, anxiety rates were 24.9% (*n* = 226) based on CAQ scores and 34.9% (*n* = 316) using NRS scores (2). A longitudinal online survey assessed emotional problems in 5,795 Brazilian children and adolescents aged 5–17 years between June to November 2020; the participants were follow-up by assessments every 15 days until June 2021. Their weighted prevalence rates of anxiety, depression, and total emotional symptoms at baseline were 29.7, 36.1 and 36%, respectively (19). Although these studies in Brazil involved different populations and slightly different methodologies, anxiety in children in this study was clearly lower than previous estimates. This reduction could be attributed to improved pandemic control in Brazil, including a decline in COVID-19-related deaths and increased vaccination rates, including for children. Additionally, the anxiety questionnaires in this study were completed face-to-face by the children themselves, unlike previous studies where responses were collected online with parental support. Online surveys have various limitations, which may have influenced previous findings.

In the current and two previous studies (2, 8), the prevalence of anxiety was higher based on CAQ scores than NRS or VAS scores. These differences may be due to the characteristics of the instruments. The CAQ measures different aspects of anxiety, such as various emotional states, whereas the NRS and VAS focus on overall anxiety levels.

It is not possible to conclude that decreased prevalence of childhood anxiety is related solely to the return to in-person classes. However, school closures during the COVID-19 pandemic had significant social implications beyond the primary educational impact, notably impacting the psychological and emotional well-being of students. Viner et al. conducted a narrative synthesis of 36 studies from 11 countries during the first wave of the COVID-19 pandemic (February–July 2020) (5). These studies, involving 79,781 children and adolescents and 18,028 parents, showed that short-term school closures, as part of broader social lockdown measures, had adverse effects on mental health (including increased anxiety and depressive symptoms) and health behaviors (e.g., lower physical activity) among children and adolescents (5).

In a systematic review of 10 studies assessing the impact of school closures on the mental and physical health of children and adolescents, Chaabane et al. (21) found that closures notably affected children with disabilities and those from lower-income families by depriving them of critical school-based services and resources. Additionally, the disruption of daily routines linked to school closures was associated with increased stress and negative emotional reactions among children. Longer durations of school closure and reduced physical activity were correlated with increases in body mass index and a higher prevalence of childhood obesity (21).

The prevalence of anxiety in the present study was higher than that reported in a study conducted in Sweden (22). In particular, among 774 Swedish children (6–14 years old) during the first wave of the COVID-19 pandemic, the prevalence of anxiety (CAQ scores >9 or NRS scores ≥7) was 2.5% (CAQ scores) and 2.7% (NRS scores). This difference is notable given that preschools and primary schools in Sweden remained open throughout the pandemic, which probably helped mitigate adverse educational, mental, and physical health effects. Attending school and participating in leisure activities with peers have been shown to affect health substantially in children (23). A qualitative study during the COVID-19 pandemic in the United Kingdom revealed that children experienced a profound sense of missing out on opportunities and found daily life without leisure activities to be monotonous (23). Research suggests that childhood anxiety may raise the risk of secondary depression and disrupt social, emotional, and academic development, potentially leading to substance abuse, suicide, educational underachievement, and functional impairment (24).

This study identified important factors contributing to anxiety among schoolchildren in Brazil during the COVID-19 pandemic. Analyses using the CAQ and VAS instruments revealed that age is significantly associated with higher anxiety levels (i.e., each additional year of age was linked to increased anxiety). When using CAQ scores of ≥9 (*p* < 0.001), the age of children was associated with higher anxiety levels. The prevalence of anxiety was higher for 12-year-olds based on CAQ scores of ≥9 (39%, *n* = 113) and for 6-year-olds based on VAS scores of >7 (63%, *n* = 31). Differences across ages might also play a role; older children may have a better understanding of the pandemic and their personal experiences, leading to more accurate self-reporting of their emotions. Further research is needed to explore these differences and clarify how accurately each tool measures anxiety.

Another factor related to anxiety was the reduction in family income reported by parents during the pandemic. However, economic factors may have negative long-term effects on children. Although we did not investigate the association between parental income and anxiety in children, economic instability increases anxiety levels among children, as suggested by previous research (1). Our findings differ from a systematic review that included 40,807 children and adolescents in pre-COVID-19 studies and 33,682 in during-COVID-19 studies. Changes in depression symptoms were most significant among mid-to high-income populations (standardized mean change [SMC], 0.35; 95% CI, 0.07–0.63) and in studies in North America (SMC, 0.25; 95% CI, 0.15–0.36) and Europe (SMC, 0.35; 95% CI, 0.17–0.53) (25).

Additionally, in this research, the use of open questions could potentially improve our understanding of the experiences and feelings of children, offering a nuanced perspective on the issue. The statements of children indicated worry and fear related to the possibility of the death of family members, parents, and pets, as well as general illness during the COVID-19 pandemic. On the other hand, children cited parents and family members as being responsible for keeping them happy and calm. Therefore, the importance of family is evident, especially during critical or extreme situations, such as a pandemic (8, 22). Furthermore, it is important to understand protective factors like emotion regulation skills. A systematic review and meta-analysis of 141 studies was conducted during the COVID-19 pandemic with 1,018,171 children and adolescents aged 6 to 17 years old. On the individual level, being male, older, living in urban area, and having a positive effect, better emotional functioning, positivity, better physical health, more physical activity, and higher quality of life were associated with less anxiety. On the family level, good family socioeconomic status, better family functioning, and more family support were significant protective factors (26).

A study that investigated the influence of the COVID-19 pandemic on growth rates of anxiety disorder found that the age group with the highest prevalence of anxiety disorders worldwide was 25–29 years, whereas the age group with the highest incidence rate was 10–14 years. Projections indicate that by 2050, the number of individuals affected by anxiety disorders may reach 87.36 million (95% UI, 59.28–115.44). It is also anticipated that the prevalence of anxiety disorders among the 15–19 age group will exceed that of other age groups by 2050 (27). Our findings highlight the importance of ongoing mental health care for children and adolescents in the post-pandemic period. Integrating soft skills—such as communication, teamwork, empathy, and professionalism—into the education of healthcare professionals is becoming increasingly important. These skills are highly valued and can play a significant role in managing mental health conditions in this population (28).

Finally, school nurses play a critical role in different activities, including during public health emergencies in school settings. However, this role needs to be improved in the Brazilian setting. Brazil is a vast and continental country. At the same time, some areas, such as the South and Southeast regions, are more developed, while others, like the North and Northeast regions, have much lower development rates, further exacerbating social inequalities. There are also notable differences between living in small towns, like the one discussed in this study, and in larger cities.

### 4.1 Limitations

This study had some limitations. Data were collected in a small city in Brazil, limiting the generalizability of the findings to all Brazilian children. We only did one measurement of childhood anxiety. We had an 89% response rate, and not all parents completed all the questions of every survey. We did not examine the impact of parents’ anxiety on children’s mental health, as reported in a study conducted in Pakistan (29). This is a crucial issue to be considered in public policy programs. Therefore, our conclusions should be interpreted with caution. Also, the children responded to the open questions with short answers inherent to their age groups and the instrument used, thus limiting in-depth qualitative analyses.

### 4.2 Implications for nursing practice

Already completing 5 years since the beginning of the pandemic, public policies should prioritize long-term actions to reduce childhood and adolescent anxiety, as many children affected by the pandemic are now adolescents with evolving needs. In addition, different age groups may have other health behaviors and needs. This research contributes to knowledge that can guide public policies through understanding the evolution of the COVID-19 pandemic in Brazil and provides a basis for comparison with literature from other countries.

## 5 Conclusion

This study demonstrated that the prevalence of anxiety among schoolchildren returning to in-person classes during the COVID-19 pandemic was 13.5% (*n* = 99) and 6.7% (*n* = 49) when using their CAQ and VAS scores, respectively. The prevalence of anxiety increased along with the children’s ages. These findings showed a lower prevalence of anxiety after 2 years than those reported at the beginning and 1 year after the COVID-19 pandemic, which further emphasize the need for public health interventions targeting different age groups.

Although this study does not offer definitive conclusions, it provides valuable insights concerning the impact of COVID-19 on Brazilian schoolchildren in a small city. For early prevention of mental health disorders, school staff and health professionals must increase awareness regarding children’s feelings of anxiety through family-based and community-based action. Moreover, new studies should evaluate anxiety among children in Brazil.

## Data Availability

The raw data supporting the conclusions of this article will be made available by the authors, without undue reservation.
